# Nationwide Distribution and Prevalence of Epizootic Haemorrhagic Disease Virus Infection in Portugal (2023–2025)

**DOI:** 10.3390/v18090936

**Published:** 2026-08-27

**Authors:** Silvia C. Barros, Fernanda Ramos, Inês S. Maia, Laura Catarino, Fábio A. Abade dos Santos, Ana M. Henriques, Ana Duarte, Margarida Dias Duarte

**Affiliations:** 1 Laboratory of Virology, Strategic Unit for Animal Production Research and Services, National Institute for Agrarian and Veterinary Research, Avenida da República, Quinta do Marquês, 2780-157 Oeiras, Portugal; fernanda.ramos@iniav.pt (F.R.); ines.maia@iniav.pt (I.S.M.); lauracatarino28@gmail.com (L.C.); fabio.abade@iniav.pt (F.A.A.d.S.); margarida.henriques@iniav.pt (A.M.H.); ana.duarte@iniav.pt (A.D.); margarida.duarte@iniav.pt (M.D.D.); 2 NOVA School of Science and Technology, Department of Life Sciences, 2829-516 Caparica, Portugal; 3 Veterinary and Animal Research Centre (CECAV), Faculty of Veterinary Medicine, Lusófona University, 1749-024 Lisbon, Portugal; 4 Centre for Interdisciplinary Research in Animal Health (CIISA), 1300-477 Lisbon, Portugal

**Keywords:** epizootic haemorrhagic disease, EHDV, cattle, deer, Portugal

## Abstract

Epizootic haemorrhagic disease virus (EHDV) is an insect-borne virus that affects ruminants. It has recently emerged as a significant threat to livestock and wildlife, particularly deer and cattle. Since its introduction into Europe in late 2022, severe outbreaks have been reported across several European countries, including Italy, Spain, France, and Portugal. The first confirmed circulation of EHDV in Portugal was reported in July 2023, involving serotype 8 (EHDV-8) in cattle from the municipalities of Moura and Barrancos, in the Beja district of the Alentejo region, alongside concurrent circulation in cervids. Here, we analyse the prevalence and spatial distribution of EHDV during the first recorded outbreaks in Portugal (2023–2024), describing its geographic spread across the country and subsequent disappearance by 2025. Higher viral loads were significantly associated with the presence of clinical signs. These findings provide new insights into the epidemiology of EHDV infection and suggest that higher viral loads are associated with the occurrence of clinical disease in naturally infected animals.

## 1. Introduction 

Epizootic haemorrhagic disease (EHD) is an infectious but non-contagious viral disease that affects wild and domestic ruminants, caused by the epizootic haemorrhagic disease virus (EHDV), a member of the *Orbivirus* genus within the *Sedoreoviridae* family.

EHDV is a non-enveloped virus with an icosahedral capsid and a segmented double-stranded RNA (dsRNA) genome comprising 10 linear segments of varying sizes, which encode seven structural proteins (VP1–VP7) and several non-structural (NS1–NS3/3a and NS4) proteins [1]. This segmented genome organisation, which is characteristic of orbiviruses, facilitates genetic reassortment during co-infection events and the generation of novel genomic constellations, thereby contributing to viral diversity and potentially enhancing adaptation to changing ecological conditions [2,3].

The outer capsid proteins, particularly VP2 and VP5, play a critical role in host cell attachment and entry, as well as in serotype specificity, while the inner core proteins are involved in genome replication and transcription. The segmented genome organisation, which is also found in other orbiviruses, enables the virus to generate new variants and adapt to changing ecological conditions.

Similar to bluetongue virus (BTV), EHDV is transmitted by hematophagous *Culicoides* midges and exhibits a marked seasonal pattern that closely follows vector activity [4,5]. The two viruses also share important ecological and epidemiological features, including overlapping vector species and host ranges, which may result in their concurrent circulation in the same areas and complicate disease surveillance and diagnosis. In cattle, signs of EHD may range from subclinical or asymptomatic infection to severe disease, with clinical signs including fever, oral lesions, oedema, hypersalivation, lameness, and, in some cases, death [5]. In small ruminants, EHDV infection is usually subclinical, showing few overt clinical signs [6]. In contrast, susceptible cervids may develop severe acute disease, characterised by fever, weakness, lameness, oedema, haemorrhagic lesions, sialorrhea, and respiratory and neurological signs. In severe cases, death may occur [7,8].

The occurrence of asymptomatic or mildly affected animals is of particular epidemiological relevance because infected animals may remain undetected while potentially contributing to the maintenance and spread of the virus through exposure to competent *Culicoides* vectors. Moreover, the clinical manifestations of EHDV are not pathognomonic and may overlap substantially with those of BTV and other diseases, making clinical differentiation difficult and highlighting the importance of laboratory-based surveillance and molecular diagnosis. Consequently, relying solely on clinical observations may lead to underdiagnosis or misinterpretation of EHDV infections, with implications for disease surveillance, control, and assessment of its potential impact.

EHD is a listed disease in the World Organisation for Animal Health (WOAH) [9,10] Terrestrial Animal Health Code and must be reported to the WOAH (source: “https://animal-diseases.efsa.europa.eu/EHDV/ (accessed on 6 May 2026)”). Historically, EHD has been predominantly reported in North America, Africa, Asia, and parts of Oceania, with sporadic incursions into the Mediterranean basin [11,12]. However, in recent years, the geographical distribution of orbiviruses has expanded, driven by factors such as climate change, globalisation, and the spread of competent vectors [9,10]. Europe has experienced repeated incursions of arboviruses of veterinary importance, particularly BTV, which demonstrates a well-established pattern of introduction from North Africa through the windborne dispersal of infected *Culicoides* midges [13]. This mechanism has facilitated the rapid spread of infection across the Mediterranean into southern Europe.

A similar epidemiological pattern has been proposed for EHDV serotype 8 (EHDV-8), which was first detected in Europe in 2022. Following initial detections in Italy [14], particularly in Sardinia and Sicily, outbreaks were reported shortly thereafter in southwestern Spain in November 2022 [15].

Molecular analyses have demonstrated a high degree of genetic similarity between European and North African strains, providing strong support for a recent introduction from Tunisia and subsequent stepwise dissemination across southern Europe mediated by infected vectors [7].

Given its geographical location and extensive land border with Spain, Portugal is particularly susceptible to the introduction of vector-borne diseases circulating on the Iberian Peninsula. This vulnerability has previously been documented in the case of bluetongue, with incursions into Portugal often following outbreaks in neighbouring Spanish regions [16].

The present study aimed to assess the nationwide prevalence of EHDV infection in Portugal following its introduction in the country. The study characterised the temporal and geographical distribution of EHDV molecularly confirmed cases and evaluated patterns of spread across the country. These findings improve our understanding of the epidemiology, evolution, and transmission dynamics of EHDV in Southwest Europe, providing valuable insights for future surveillance and control strategies in regions at risk of arboviral emergence.

## 2. Materials and Methods

### 2.1. Study Population

The study population comprised 5293 cattle, 15 goats, 53 sheep, and 20 deer originating from various regions of Portugal. Between 2023 and 2025, blood samples from cattle, goats, and sheep and blood and organs from deer were received at the National Institute for Agrarian and Veterinary Research (INIAV, I.P.) for molecular diagnosis of EHDV. Cattle samples were submitted either due to clinical suspicion of EHDV infection or for export certification purposes.

### 2.2. Molecular Diagnosis

Total nucleic acids were extracted from 200 μL of blood or tissue samples using the IndiMag^®^ Pathogen Kit (Indical, Leipzig, Germany) in a KingFisher Flex extractor (Thermo Fisher Scientific, Waltham, MA, USA), following the manufacturer’s instructions. The extraction procedure was validated by RT-qPCR detection of a spiked synthetic RNA control (VLP-RNA EXTRACTION; Meridian Life Science, Memphis, TN, USA), which was added to each sample (4 μL per sample) prior to extraction. A negative extraction control, consisting of a sample processed in parallel without biological material, was included throughout the procedure.

Samples were screened for EHDV using a previously described RT-qPCR assay [17], and serotype 8 was identified using a specific RT-qPCR assay [14]. Both assays were performed using the One-step NZYSpeedy RT-qPCR Probe Kit (NZYtech, Lisbon, Portugal) in a final reaction volume of 20 μL. Amplification was carried out on a Bio-Rad CFX96™ Real-Time PCR Detection System (Bio-Rad Laboratories, Hercules, CA, USA).

### 2.3. EHDV RNA RT-qPCR Quantification Curve

To construct the calibration curve that correlates RT-qPCR quantification cycle (Cq) values with EHDV RNA copy numbers, an in vitro-transcribed RNA fragment corresponding to the genomic region targeted by the EHDV-specific RT-qPCR assay described by Maan et al. (2017) was generated and used as a quantification standard [18]. This fragment was then amplified and subsequently purified using the QIAquick Gel Extraction Kit (Qiagen, Hilden, Germany). The purified amplicon was cloned into the pCR2.1 vector using One Shot TOP10 chemically competent Escherichia coli cells (Invitrogen, Thermo Fisher Scientific, Waltham, MA, USA). Plasmid recombinant DNA was extracted using the NZYMiniprep kit (NZYTech, Lisbon, Portugal) and quantified using the Qubit 1X dsDNA HS Assay kit and the QubitTM 4 Fluorometer (Thermo Fisher Scientific, Waltham, MA, USA). In vitro transcription was performed using the MEGAscript T7 Transcription Kit (Thermo Fisher Scientific, Waltham, MA, USA) according to the manufacturer’s instructions. The transcribed RNA was then treated with TURBO DNase (Thermo Fisher Scientific, Waltham, MA, USA) to remove residual plasmid DNA, after which it was purified and tested by PCR to confirm the absence of DNA. The RNA concentration was also determined using the QubitTM 4 Fluorometer and the Qubit RNA BR assay kit. The corresponding RNA copy number was calculated based on the transcript length and molecular weight. Ten-fold serial dilutions of the transcribed RNA, covering the expected dynamic range of the assay, were prepared and analysed by RT-qPCR in triplicate. A calibration curve was generated by plotting the obtained Cq values against the base 10 logarithm of the calculated RNA copy number. Linear regression analysis was then used to determine efficiency, linearity, and correlation of the assay.

### 2.4. Statistical Analysis

Viral loads were extrapolated from the RT-qPCR Cq values using the standard calibration curve equation log_10_(viral load) = (37.588 − Cq)/3.297 and were subsequently log_10_-transformed for analysis. Because the Shapiro–Wilk test indicated a significant departure from normality (*p* < 0.001), non-parametric methods were used throughout. Differences in viral load between symptomatic and asymptomatic animals were assessed using the Mann–Whitney U test, while differences among host species and geographical regions were evaluated using the Kruskal–Wallis test. When the Kruskal–Wallis test indicated a significant overall effect, pairwise comparisons were performed using Dunn’s post hoc test, with *p*-values adjusted for multiple comparisons using the Bonferroni correction. Statistical significance was set at α = 0.05. All statistical analyses were performed in Python 3.14.7 (Python Software Foundation), using the SciPy (v 1.18.1) and scikit-posthocs (v 0.14.0) packages [17].

## 3. Results

**Figure 1:**
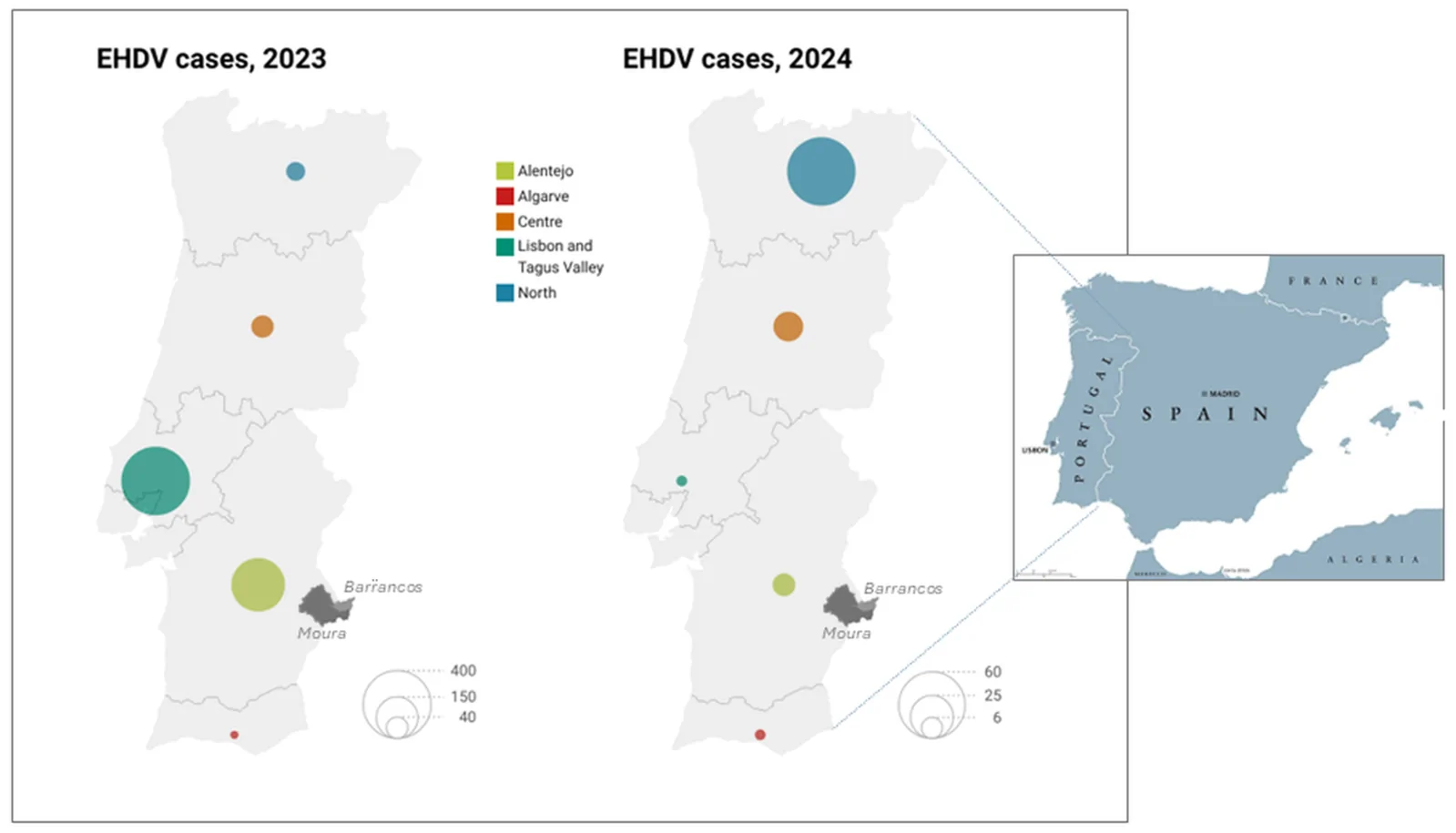
Distribution of EHDV cases in mainland Portugal, 2023–2024. The size of the circles is proportional to the number of reported cases in each region.

On 19 July 2023, whole blood samples from two cattle presenting clinical signs consistent with epizootic haemorrhagic disease were submitted to INIAV for diagnosis. Clinical signs included fever, lesions of the oral and nasal mucosa, tongue oedema with dark discoloration, hypersalivation, and lameness. The two bovines originated from the municipalities of Moura and Barrancos (Beja district), in the Alentejo region (Figure 1). Subsequent RT-qPCR analyses confirmed infection with epizootic haemorrhagic disease virus serotype 8 (EHDV-8), constituting the first reported occurrence of the disease in Portugal.

Following this initial detection, the virus spread progressively across the country from July to December of the same year.

In 2023 and in the following years, a total of 5293 cattle, 15 goats, 53 sheep, and 20 wild cervids originating from 2708 holdings across different regions of Portugal were tested by RT-qPCR.

EHDV RNA was detected in cattle and wild cervids, whereas all sheep and goats, tested in the context of intra-community or as part of differential diagnosis with BTV, were negative (Table 1). However, the limited sample sizes for these species warrant caution in interpreting the absence of EHDV RNA detection.

The percentage of cattle testing EHDV RNA-positive declined markedly over the studied period, with a greater than fourfold reduction from 22.5% in 2023 to 4.84% in 2024, reaching 0% in 2025 (Table 1). A similar, albeit more pronounced, temporal trend was observed in deer, with positivity peaking at 60% in 2023 and no positive animals identified in subsequent years.

Viral load analysis was performed on a subset of 682 RT-qPCR-positive animals (69 symptomatic and 613 asymptomatic) selected from the overall study population. This subset included only samples analysed under identical RT-qPCR conditions, including the same amplification kit and protocol, to ensure that viral loads extrapolated from Cq values were directly comparable. Symptomatic animals had significantly higher viral loads than asymptomatic animals, with median values of 11,908 and 1951 viral copies, respectively. A Mann–Whitney U test confirmed a statistically significant difference between the two groups (U = 13,316, *p* = 4.47 × 10^−7^).

All red deer, roe deer, and mouflon positive samples corresponded to fatal cases of EHDV infection. The assessment of clinical signs and macroscopic lesions in wild cervids was not possible, as the samples were received by the laboratory after collection and were not accompanied by detailed clinical or necropsy records.

**Figure 2:**
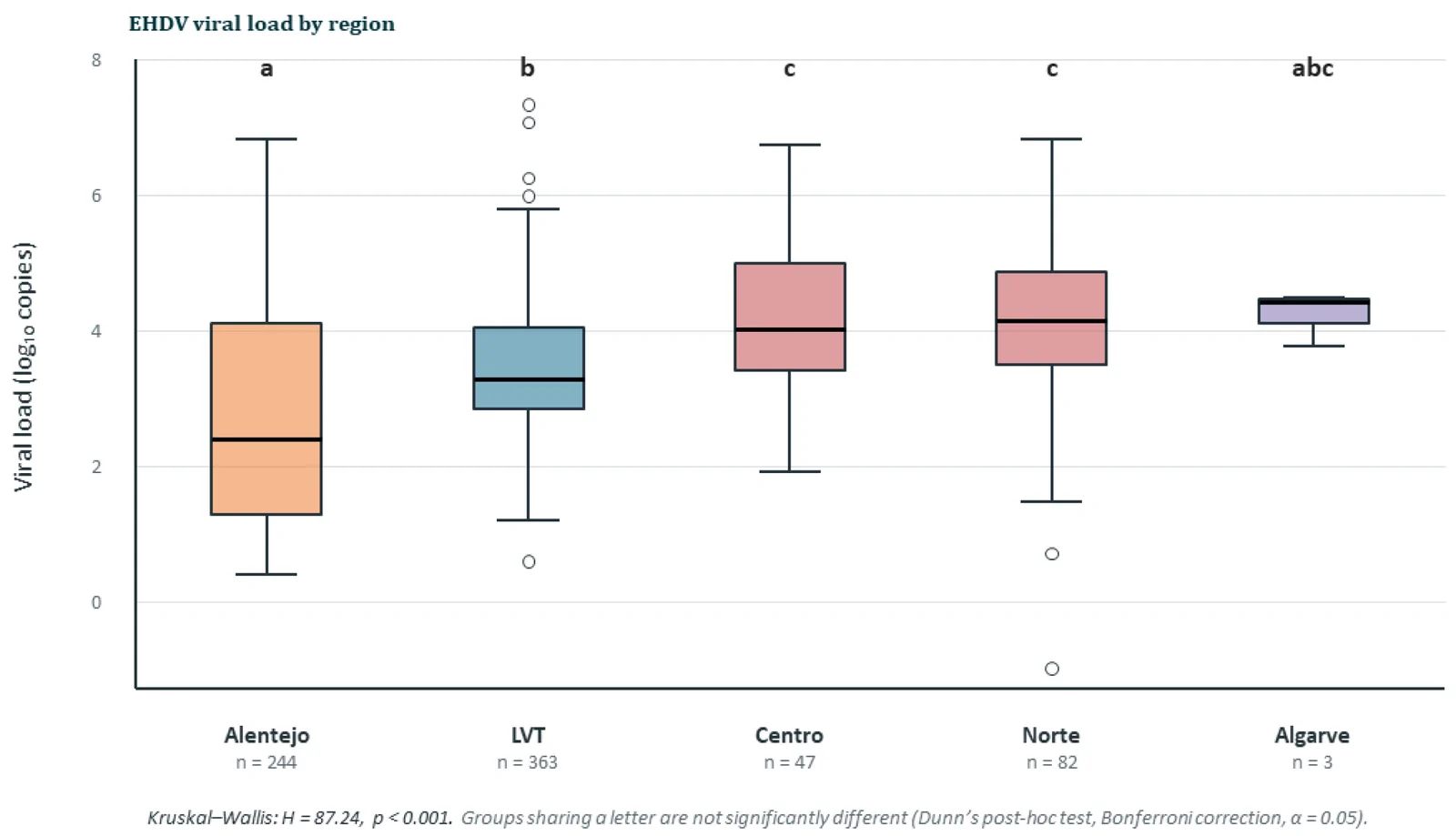
Regional distribution of EHDV viral loads in naturally infected animals sampled across mainland Portugal (2023–2024). Samples are grouped by NUTS II region of origin (*x*-axis): Alentejo (n = 244), Lisbon and Tagus Valley (LVT; *n* = 363), Centro (*n* = 47), Norte (*n* = 82), and Algarve (*n* = 3). Viral loads differed significantly among regions (Kruskal–Wallis test: H = 89.33, *p* < 0.001), and pairwise differences were assessed using Dunn’s post hoc test with Bonferroni correction for multiple comparisons. Regions that do not share a common letter above the boxes or color differ significantly (α = 0.05).

**Figure 3:**
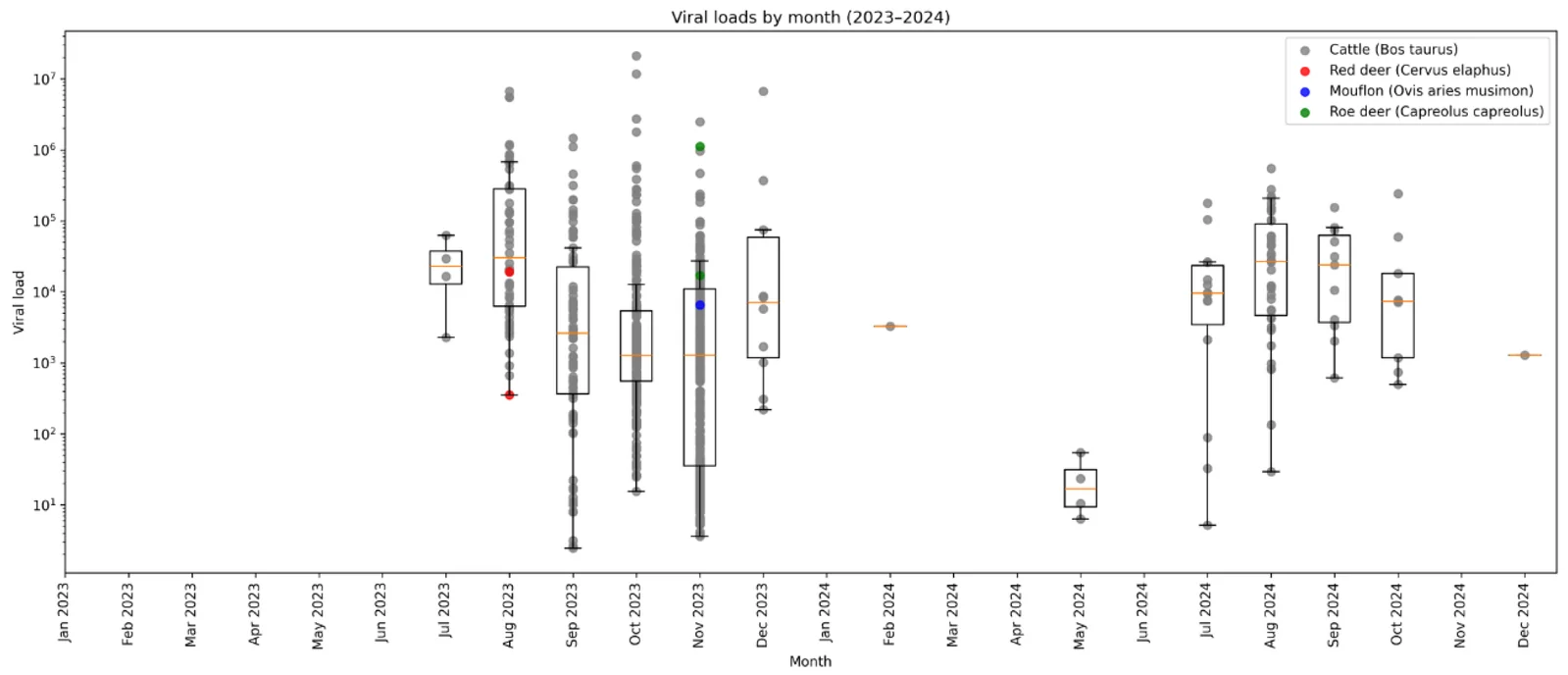
Temporal dynamics of viral loads in cattle and cervids sampled between January 2023 and December 2024. Viral loads were extrapolated from RT-qPCR Cq values using a standard calibration curve. Individual viral load measurements are shown as points plotted against the month of sampling. The *x*-axis includes all months within the study period, regardless of sampling activity, and the *y*-axis represents viral load on a log_10_ scale. Grey circles correspond to cattle (*Bos taurus*), red to red deer (*Cervus elaphus*), blue to mouflon (*Ovis aries musimon*), and green to fallow deer (*Dama dama*). Monthly boxplots summarise the distribution of viral loads, with the central line indicating the median, the boxes representing the interquartile range (IQR), and the whiskers extending to 1.5 × IQR. The figure illustrates the temporal variation in viral loads and the contribution of domestic and wild ruminant hosts over the course of the study.

In 2023, most cases were recorded in the central and southern regions of Portugal, with particularly high numbers in the Lisbon and Tagus Valley (*n* = 410) and Alentejo (*n* = 246) NUTS II regions, where cattle density is higher. In contrast, the North region reported a comparatively low number of cases (*n* = 25) during this year (Table 2). In 2024, a marked shift in the geographical distribution was observed, with the North region accounting for the highest number of cases (*n* = 64), while all other regions experienced a substantial decline, reporting only minimal figures (Table 2, Figure 2 and Figure 3). Samples for which Cq values were converted to viral loads originated from LVT (*n* = 363), Alentejo (*n* = 245), Norte (*n* = 82), Centro (*n* = 46), and Algarve (*n* = 3). The corresponding median viral loads were 1.897 × 10^3^, 2.720 × 10^3^, 1.370 × 10^4^, 1.088 × 10^4^, and 2.658 × 10^4^ viral copies, respectively. However, the estimate for the Algarve region should be interpreted with caution due to the limited number of samples. Viral loads differed significantly among geographical regions (Kruskal–Wallis test: H = 89.33, *p* = 1.83 × 10^−18^), indicating that viral burden varies across regions. Viral loads differed significantly among geographical regions (Kruskal–Wallis test: H = 89.33, *p* < 0.001), with Dunn’s post hoc test (Bonferroni-corrected) revealing significantly higher viral loads in the Centro and Norte regions than in both Alentejo and LVT (*p* < 0.001), while Centro and Norte did not differ from each other, and the Algarve estimate remained inconclusive owing to its small sample size (Figure 2). These differences may reflect regional variation in transmission dynamics, host population characteristics, environmental conditions, or sampling strategies.

Positive samples were detected from July to December in the first year and from July to October in the second year (Figure 3). This temporal distribution, consistent with patterns previously described for BTV circulation, closely mirrors the seasonal dynamics of *Culicoides* spp. vectors, whose activity generally increases during the warmer months and declines as temperatures decrease in late autumn. The positive animals identified in the Azores are likely associated with the importation of animals from mainland Portugal rather than resulting from autochthonous case transmission.

Analysis of viral load by host species was heavily skewed toward cattle (*Bos taurus*; *n* = 736), with only five samples originating from wildlife species (roe deer, red deer, and mouflon). No significant differences in viral load were detected among host species (Kruskal–Wallis test: H = 3.53, *p* = 0.318).

The available data indicate that, despite the morbidity associated with EHDV infection in the cattle included in this study, no mortality was reported to our knowledge.

## 4. Discussion

Molecular techniques, particularly real-time RT-PCR, are highly sensitive and specific for identifying infected animals, which supports the timely implementation of surveillance and control measures. In cattle, which can act as important amplifying hosts, molecular detection helps to understand how the virus is transmitted and how the disease spreads geographically. Furthermore, laboratory confirmation is essential for distinguishing EHDV infection from other clinically similar orbiviral diseases, such as bluetongue, thereby improving diagnostic accuracy and informing appropriate animal health responses [6]. Molecular surveillance also plays a crucial role in monitoring the persistence and seasonal circulation of viral strains, as well as their potential emergence, supporting risk assessment and disease management strategies at both national and regional levels.

Out of the 802 cattle that tested EHDV RNA-positive, 516 (64.3%) samples were obtained from asymptomatic animals, tested in the context of export procedures, indicating that a large proportion of infections may frequently occur in the absence of clinical signs and remain undetected in the absence of active surveillance. Historically, EHDV infection in cattle has been considered predominantly asymptomatic or subclinical, although clinical disease may occur depending on the circulating strain and host factors [19].

Symptomatic animals accounted for 35.7% (286/802) of the animals included in the study. The clinical signs reported by field veterinarians were consistent with those previously described in cattle infected with EHDV and included fever (up to 40.5 °C), lameness frequently associated with laminitis and hoof pain, sialorrhea, rhinorrhoea, oral erosive ulcerative lesions of the nasal planum, and tongue oedema. These manifestations largely reflect the vascular injury and inflammatory processes induced by the virus and show considerable overlap with the clinical presentation of bluetongue, making laboratory confirmation essential for differential diagnosis. Differences in viral load among host species have been suggested for EHDV, with available evidence indicating that cervids, particularly white-tailed deer, are generally more susceptible and may develop higher levels and more prolonged viraemia than cattle [12]. In contrast, cattle infections are often subclinical or mild, despite the presence of detectable viraemia, suggesting a different host–pathogen interaction compared with highly susceptible cervid species [20,21]. However, data from naturally infected populations remain limited, and substantial variability is observed depending on viral strain, host factors, and stage of infection, making direct comparisons between species difficult to generalise. Although viral loads were determined for a large proportion of the samples analysed in the present study, no link was found between host species and higher viral loads. This was primarily due to the extremely limited number of samples obtained from wild cervids, which prevented meaningful comparisons with cattle and robust conclusions regarding species-specific differences in viraemia. Furthermore, as the wild cervid included in this study was not examined for clinical signs or macroscopic lesions at the time of death, other potential causes of death cannot be excluded, representing a limitation of the present study. Despite being unable to assess species-specific differences in viral load, our analysis revealed a clear association between viral burden and the occurrence of clinical disease. Symptomatic animals (*n* = 69) had significantly higher viral loads than asymptomatic animals (*n* = 613), with median values of 11,908 and 1951 viral copies, respectively. This difference was highly significant (Mann–Whitney U test: U = 13,316, *p* = 4.47 × 10^−7^), indicating that animals displaying clinical signs generally harboured greater amounts of circulating viral RNA, therefore suggesting an association between higher viral loads and the presence of clinical signs. While these results do not prove that viral replication causes disease severity, they support the idea that increased viral replication leads to the development of clinical signs. Similar associations between viremia and disease severity have been reported for orbiviral infections, particularly BTV, where higher viral loads are often associated with more severe clinical outcomes. However, host immune responses, viral virulence, and the timing of sample collection are also recognised as important determinants of disease manifestation [22,23].

The first cases of EHDV in Portugal, confirmed by RT-qPCR, were detected in mid-July 2023 in cattle from the municipalities of Moura and Barrancos (Beja district), located in the Alentejo region near the Spanish border.

Notably, the Beja district coincides with the location where the first outbreak of BTV was reported in Portugal in 2004 [16], highlighting its historical role as a potential entry point for orbiviral diseases in the country. This pattern may be associated with the region’s important livestock production system, the high density of cattle herds, its proximity to the Spanish border, or a combination of these factors.

As cattle, the deer originated from border regions along the Portuguese–Spanish frontier. Following its introduction, EHDV circulated in Portugal during two consecutive transmission seasons, namely the summer–autumn periods of 2023 and 2024, with no cases reported in 2025.

A marked reduction in the number of reported cases was observed between the two years, decreasing from 725 in 2023 to 83 in 2024, suggesting a substantial decline in virus circulation (Table 1). This reduction was particularly evident in the Lisbon and Tagus Valley regions, which, despite accounting for the highest number of detections in 2023, experienced a sharp decrease to only one case in 2024. The observed reduction in virus detection may, at least in part, reflect a decrease in the number of animals tested for export purposes, as well as changes in the exportation requirements imposed by destination countries. Given that a substantial proportion of infected animals are asymptomatic, reduced testing intensity can lead to an underestimation of virus circulation.

In contrast, small ruminants, including sheep and goats, consistently tested negative throughout the study period, despite being included in the sampling strategy. No significant differences in viral load were detected among host species (Kruskal–Wallis test: H = 3.53, *p* = 0.318). However, the relatively low number of samples collected from these domestic species limits the interpretation of the negative findings and precludes ruling out EHDV circulation in small ruminant populations under the epidemiological conditions studied. Similarly, the marked imbalance in sample size, particularly the very limited representation of wild cervids, substantially reduced the statistical power of the analysis and prevents reliable assessment of species-specific differences in viral load. Consequently, the absence of significant differences should be interpreted with caution, as it may partly reflect the uneven and limited sampling across host species.

In both years, positive samples were first detected during summer; however, detection persisted until December in the first year and until October in the second year (Figure 3). This seasonal pattern closely reflects the dynamics of *Culicoides* spp. vectors. The increased abundance and feeding activity of these hematophagous midges during summer and early autumn create optimal conditions for virus transmission among susceptible hosts. In contrast, the lower temperatures observed in winter reduce vector survival, reproduction, and flight activity, thereby constraining viral spread [10,24]. These results highlight the strong association between EHDV circulation and vector ecology, underscoring the critical role of climate and seasonality in shaping the epidemiology of orbiviral diseases in Portugal.

Most bluetongue outbreaks in Europe have been directly linked to North Africa, driven by the windborne dispersal of BTV-infected *Culicoides* midges from this region. A similar introduction pathway has been described for EHDV-8, following its initial emergence in Italy in 2022, likely originating from North Africa [14]. Shortly thereafter, the first Spanish outbreaks were reported in southwestern Spain (Andalusia) in late 2022, subsequent to detections in Sardinia and Sicily. Notably, EHDV-8 strains circulating in Spain show >99–99.9% genetic identity with those identified in Tunisia in 2021, supporting a North African origin and a stepwise spread mediated by windborne *Culicoides* midges from North Africa to Italy and subsequently to Spain [15,25].

Given its geographic proximity and shared border with Spain, Portugal is particularly susceptible to the incursion of arboviruses transmitted by *Culicoides* spp., as previously observed for BTV [16]. In this context, the introduction and subsequent spread of EHDV-8 into Portugal were not unexpected following its rapid dissemination across southwestern Spain. Similarly, the first EHDV-8 case in France was detected two months later, in September 2023, in the Pyrénées-Atlantiques region along the Spanish border [26]. This epidemiological pattern further reinforces the role of cross-border movement of infected vectors, particularly through windborne dispersal, in shaping the transmission dynamics of orbiviral diseases across the Iberian Peninsula and into neighbouring regions.

The circulation of EHDV in Portugal in 2024 overlapped with that of bluetongue virus serotype 3 (BTV-3) during the final two months of EHDV detection. BTV-3 was introduced into the country in September 2024 and had a significant epidemiological impact that year [27]. The co-circulation of multiple orbiviruses within the same ecological and epidemiological context raises the possibility of competitive interactions affecting their transmission dynamics. Indeed, arboviruses sharing common vectors and hosts may interact through mechanisms such as viral interference, competition for cellular resources, or vector-mediated effects, ultimately influencing their relative fitness and transmission efficiency. Experimental studies have demonstrated that co-infection of arthropod vectors with different viruses can result in antagonistic interactions, where one virus may suppress or outcompete another, thereby shaping patterns of transmission [28].

In this context, the introduction of a new bluetongue virus serotype (BTV-8) in 2025 may have contributed to the apparent disappearance of EHDV circulation, potentially through competitive exclusion or interference mechanisms within shared *Culicoides* spp. vector populations or ruminant hosts [28]. Although direct evidence of such interactions between EHDV and BTV remains limited, the co-circulation of multiple arboviruses and their potential for interaction is increasingly recognised as an important factor influencing the epidemiology of vector-borne diseases, warranting further investigation.

## Figures and Tables

**Table 1:** Number of samples tested for EHDV-RNA from cattle, wild cervids (red deer, roe deer, and mouflon), sheep, and goats in the triennium 2023–2025.

Year	No. Cattle Positive/Tested	No. Wild Cervids Positive/Tested	No. Sheep Positive/Tested	No. Goat Positive/Tested	Total Positive/Tested	No. Positive Holdings
2023	719/3194 (22.5%)	6/10 (60%)	0/15 (0%)	0/10 (0%)	725/3229 (22.5%)	2642
2024	83/1713 (4.84%)	0/9 (0%)	0/19 (0%)	0(0%)	83/1741 (4.84%)	66
2025	0/386 (0%)	0/1 (0%)	0/19 (0%)	0/5 (0%)	0/411 (0%)	0
Total	802/5293 (15.15%)	6/20 (30%)	0/53 (0%)	0/15 (0%)	808/5381 (15%)	2708

**Table 2:** Regional distribution of EHDV-positive cases in Portugal by year (2023–2025). Percentages shown in parentheses represent the proportion of positive animals among those tested.

Year	Azores Positive/Tested	Alentejo Positive/Tested	Algarve Positive/Tested	Centre Positive/Tested	Lisbon and Tagus Valley Positive/Tested	North Positive/Tested	Total Positive/Tested
2023	4/27 (14.8%)	246/1662 (14.8%)	3/13 (23.1%)	37/118 (31.4%)	410/1381 (29.7%)	25/28 (89.3%)	725/3229 (22.5%)
2024	0	6/823 (0.73%)	1/19 (5.26%)	11/215 (5.11%)	1/588 (0.17%)	64/96 (66.6%)	83/1741 (4.84%)
2025	0	0/58 (0%)	0/0 (0%)	0/70 (0%)	0/230 (0%)	0/53 (0%)	0/411 (0%)
Total	4/27 (14.8%)	252/2543 (9.9%)	4/32 (12.5%)	48/403 (11.9%)	411/2199 (18.7%)	89/177 (50.3%)	808/5381 (15%)

## Data Availability

The data supporting the results of this study can be obtained by contacting the authors.

## Abbreviations

The following abbreviations are used in this manuscript:

EHDV	Epizootic Haemorrhagic Disease Virus
EHDV-8	Epizootic Haemorrhagic Disease Virus Serotype 8
EHD	Epizootic Haemorrhagic Disease
BTV	Bluetongue Virus
INIAV	National Institute for Agrarian and Veterinary Research
Cq	Quantification Cycle
HS	High Sensitivity
BR	Broad Range
NUTS II	Nomenclature of Territorial Units for Statistics level II
LVT	Lisbon and Tagus Valley
IQR	Interquartile Range
BTV-3	Bluetongue Virus Serotype 3
BTV-8	Bluetongue Virus Serotype 8

## References

[1] Forzan M., Maan S., Mazzei M., Belaganahalli M.N., Bonuccelli L., Calamari M., Carrozza M.L., Cappello V., Di Luca M., Bandecchi P. (2016). Generation of Virus like Particles for Epizootic Hemorrhagic Disease Virus. Res. Vet. Sci..

[2] McDonald S.M., Nelson M.I., Turner P.E., Patton J.T. (2016). Reassortment in Segmented RNA Viruses: Mechanisms and Outcomes. Nat. Rev. Microbiol..

[3] Gorman B.M. (1983). On the evolution of orbiviruses. Intervirology.

[4] Quaglia M., Pinoni C., Florio S., D’alessio S.G., De Ascentis M., Castilletti C., Gobbi F.G., Foxi C., Satta G., Cabras P. (2025). Transmission Routes of Oropouche Virus: Potential Role of European Biting Midges and First Oral Infection Attempt in Wild-Caught Culicoides (Subgenus Avaritia). Vet. Ital..

[5] Savini G., Afonso A., Mellor P., Aradaib I., Yadin H., Sanaa M., Wilson W., Monaco F., Domingo M. (2011). Epizootic Haemorragic Disease. Res. Vet. Sci..

[6] World Organisation for Animal Health (WOAH) (2021). Epizootic Haemorrhagic Disease (Infection with Epizootic Haemorrhagic Disease Virus). Terrestrial Manual.

[7] Muñoz-Fernández L., Agulló-Ros I., Cano-Terriza D., Abo-Zakaib-Ali F., Martínez R., Frías M., Ortiz J.A., Gortázar C., Armillotta G., Mercante M.T. (2026). Unveiling the Clinical Signs and Pathology in Red Deer (*Cervus elaphus*) Naturally Infected with Epizootic Haemorrhagic Disease Virus Serotype 8. Vet. Res..

[8] Morel M., Nederlof R.A., Espinosa-Cerrato J., Bakker J., Prieto-Yerro P., Gómez-Guillamón Manrique F., Agüero Garcia M., Talavera-Navarrete V., Camacho-Sillero L.N. (2026). Epizootic Haemorrhagic Disease Virus (EHDV) Infection in Red Deer (*Cervus elaphus*), Fallow Deer (*Dama dama*) and Mouflon (*Ovis orientalis musimon*) in South-Eastern Spain: Implications for Wildlife Health and Ruminant Disease Ecology. Animals.

[9] Hudson A.R., McGregor B.L., Shults P., England M., Silbernagel C., Mayo C., Carpenter M., Sherman T.J., Cohnstaedt L.W. (2023). Culicoides-Borne Orbivirus Epidemiology in a Changing Climate. J. Med. Entomol..

[10] Purse B.V., Carpenter S., Venter G.J., Bellis G., Mullens B.A. (2015). Bionomics of Temperate and Tropical Culicoides Midges: Knowledge Gaps and Consequences for Transmission of Culicoides-Borne Viruses. Annu. Rev. Entomol..

[11] Golender N., Hoffmann B. (2024). The Molecular Epidemiology of Epizootic Hemorrhagic Disease Viruses Identified in Israel between 2015 and 2023. Epidemiologia.

[12] Jiménez-Cabello L., Utrilla-Trigo S., Lorenzo G., Ortego J., Calvo-Pinilla E. (2023). Epizootic Hemorrhagic Disease Virus: Current Knowledge and Emerging Perspectives. Microorganisms.

[13] Purse B.V., Mellor P.S., Rogers D.J., Samuel A.R., Mertens P.P.C., Baylis M. (2005). Climate Change and the Recent Emergence of Bluetongue in Europe. Nat. Rev. Microbiol..

[14] Lorusso A., Cappai S., Loi F., Pinna L., Ruiu A., Puggioni G., Guercio A., Purpari G., Vicari D., Sghaier S. (2023). Epizootic Hemorrhagic Disease Virus Serotype 8, Italy, 2022. Emerg. Infect. Dis..

[15] Ruiz-Fons F., García-Bocanegra I., Valero M., Cuadrado-Matías R., Relimpio D., Martínez R., Baz-Flores S., Gonzálvez M., Cano-Terriza D., Ortiz J.A. (2024). Emergence of Epizootic Hemorrhagic Disease in Red Deer (*Cervus elaphus*), Spain, 2022. Vet. Microbiol..

[16] Barros S.C., Ramos F., Luís T.M., Vaz A., Duarte M., Henriques M., Cruz B., Fevereiro M. (2007). Molecular Epidemiology of Bluetongue Virus in Portugal during 2004–2006 Outbreak. Vet. Microbiol..

[17] De Leeuw I., Villalba R., Aguëro M., Mostin L., De Regge N. (2025). Influence of Inoculation Dose and Route on EHDV-8 Distribution and the Induced Immune Response in Experimentally Infected Cattle. Vet. Res..

[18] Maan N.S., Maan S., Potgieter A.C., Wright I.M., Belaganahalli M., Mertens P.P.C. (2017). Development of Real-Time RT-PCR Assays for Detection and Typing of Epizootic Haemorrhagic Disease Virus. Transbound. Emerg. Dis..

[19] Ruder M.G., Howerth E.W., Batten C. (2024). Recognition of Field Signs, Necropsy Procedures, and Evaluation of Macroscopic Lesions of Cervids Infected with Epizootic Hemorrhagic Disease Virus. Epizootic Hemorrhagic Disease Virus: Methods and Protocols.

[20] Noronha L.E., Cohnstaedt L.W., Richt J.A., Wilson W.C. (2021). Perspectives on the Changing Landscape of Epizootic Hemorrhagic Disease Virus Control. Viruses.

[21] Maclachlan N.J., Mayo C.E., Daniels P.W., Savini G., Zientara S., Gibbs E.P.J. (2015). Bluetongue. Sci. Tech. Rev..

[22] Herder V., Caporale M., MacLean O.A., Pintus D., Huang X., Nomikou K., Palmalux N., Nichols J., Scivoli R., Boutell C. (2024). Correlates of Disease Severity in Bluetongue as a Model of Acute Arbovirus Infection. PLoS Pathog..

[23] EFSA Panel on Animal Health and Welfare (2017). Bluetongue: Control, Surveillance and Safe Movement of Animals. EFSA J..

[24] Díaz-Cao J.M., López-Lorenzo G., López-Novo C., Díaz P., Remesar S., López C., Morrondo P., Fernández G., Prieto A. (2025). Description of the Clinical Findings Associated with the Epizootic Hemorrhagic Disease in Cattle from Northwestern Spain During the Emergence. Transbound. Emerg. Dis..

[25] Gondard M., Postic L., Garin E., Turpaud M., Vorimore F., Ngwa-Mbot D., Tran M.L., Hoffmann B., Warembourg C., Savini G. (2024). Exceptional Bluetongue Virus (BTV) and Epizootic Hemorrhagic Disease Virus (EHDV) Circulation in France in 2023. Virus Res..

[26] Barros S.C., Henriques A.M., Ramos F., Luís T., Fagulha T., Magalhães A., Caetano I., Abade dos Santos F., Correia F.O., Santana C.C. (2024). Emergence of Bluetongue Virus Serotype 3 in Portugal (2024). Viruses.

[27] de Oliveira M.E.d.S.P., Krokovsky L., Couto M.J.B., Guedes D.R.D., Diniz G.T.N., Ayres C.F.J., Paiva M.H.S. (2025). Evaluation of the Impact of Coinfection and Superinfection on Chikungunya and Mayaro Viruses’ Replication in Aedes Aegypti. Microorganisms.

[28] EL Hussein A., Ramig R.F., Holbrook F.R., Beaty B.J. (1989). Asynchronous Mixed Infection of Culicoides Variipennis with Bluetongue Virus Serotypes 10 and 17. J. General. Virol..

